# Association of Prostate Imaging Reporting and Data System (PI-RADS) Score and Prostate-Specific Antigen Density (PSAD) With Prostate Biopsy Histology Outcomes: A Retrospective Study

**DOI:** 10.7759/cureus.97745

**Published:** 2025-11-25

**Authors:** Vignesh Balasubaramaniam, Anurag Agarwal, Ashok Kailasa, Nurul Aimi B Ismail, Daniel Grogan, Arran Shoker, Katherine V Rhodes, Krassen Donev, Shanmugasigamani Kannan

**Affiliations:** 1 Surgery, Betsi Cadwaladr University Health Board, Bangor, GBR; 2 General Surgery, Betsi Cadwaladr University Health Board, Bangor, GBR; 3 Urology, Betsi Cadwaladr University Health Board, Bangor, GBR; 4 General Surgery, Ysbyty Gwynedd, Bangor, GBR

**Keywords:** magnetic resonance imaging, neoplasm grading, prostate histology, prostate neoplasm, prostate-specific antigen

## Abstract

Objective: This study aimed to investigate the association of Prostate Imaging Reporting and Data System (PI-RADS) and prostate-specific antigen density (PSAD) with prostate biopsy histology outcomes in a retrospective cohort.

Methods: We audited 594 consecutive men who underwent local anaesthetic transperineal prostate biopsy between January 2022 and June 2025. Data collected included age, prostate-specific antigen (PSA), prostate volume, PI-RADS score, and Gleason grade. PI-RADS scores were categorised as low (1-2), intermediate (3), and high (4-5). PSAD was categorised as <0.15 ng/mL^2^ and ≥0.15 ng/mL^2^. Multinomial logistic regression was performed to assess the association between PSAD and PI-RADS with benign histology, Gleason 6 cancers, and Gleason ≥7 cancers. Significance was set at p<0.05.

Results: The median patient age was 68.7 years, median PSA 8.20 ng/mL, and median PSAD 0.17 ng/mL^2^. About 30.1% of biopsies were benign, with malignancies in 69.9% (34.7% Gleason 6; 65.3% Gleason ≥7). High PSAD (≥0.15 ng/mL^2^) and high PI-RADS (4-5) independently are strongly associated with Gleason ≥7 cancers (OR 5.63 and 95% CI 3.54-8.96; OR 9.14 and 95% CI 4.36-19.19; both p<0.001). PI-RADS 3 demonstrated non-significant trends. Age was an independent predictor of clinically significant cancer but not Gleason 1 disease (OR 1.04; 95% CI 1.01-1.07; p=0.01)

Conclusions: Elevated PSAD and high PI-RADS scores are strongly associated with Gleason ≥7 cancers. PSAD also adds discriminatory value in PI-RADS 3 lesions.

## Introduction

Prostate cancer (PC) is the second most commonly diagnosed cancer and the fifth leading cause of cancer death among men worldwide [1]. In the United Kingdom alone, over 56,000 men are diagnosed with PC each year [2]. PC incidence rises sharply with age, yet many tumours remain indolent and asymptomatic. Consequently, clinical symptoms and diagnosis often occur in older men if they occur at all, and a substantial proportion of men with PC will die from unrelated causes without ever experiencing PC-related symptoms [3].

In clinical practice, suspicion of PC is typically based on a combination of prostate-specific antigen (PSA) testing, digital rectal examination (DRE), and imaging findings, with definitive diagnosis obtained via biopsy. Modern imaging and biomarker tools have improved the detection and risk stratification of clinically significant prostate cancer (csPCa). Multiparametric MRI (mpMRI) of the prostate is now frequently used as an advanced diagnostic modality to identify PC lesions. When interpreted using the Prostate Imaging Reporting and Data System (PI-RADS), mpMRI can accurately localise index cancer lesions in up to 92% of cases [4]. Another valuable metric is prostate-specific antigen density (PSAD), defined as the serum PSA level divided by prostate volume. The European Association of Urology (EAU) guidelines suggest that a PSAD above approximately 0.10-0.15 ng/mL^2^ is highly suggestive of PC risk. In contrast, a very low PSAD (below about 0.10 ng/mL^2^) is associated with a reduced likelihood of csPCa [5]. 

Evidence indicates that higher PI-RADS scores and elevated PSAD are strongly linked to the presence of csPCa on biopsy [6,7]. When considered together, PI-RADS scoring and PSAD appear to complement each other, offering a means to improve both "rule-in" and "rule-out" decisions for prostate biopsy [8,9]. Prostate biopsies have complications, and the ability of these parameters to assist in decision-making can potentially reduce the rate of overdiagnosis and the need for patients to undergo this procedure. Hence, this study aims to evaluate the association of PI-RADS score and PSAD with prostate biopsy histology findings.

## Materials and methods

Study design

This retrospective audit included patients who underwent local anaesthesia transperineal biopsy (LATP) at Ysbyty Gwynedd, a district general hospital in the United Kingdom. This study was registered as an audit at Ysbyty Gwynedd, Bangor, UK (approval number: 2291), and there were no alterations to clinical pathways or standard patient care. Relevant data were obtained from outpatient clinic letters, multidisciplinary outcome sheets, and the online portal. Inclusion criteria comprised all LATP procedures from January 1, 2022, to June 30, 2025, in patients with no history of PC. Exclusion criteria were the absence of an MRI or a documented PI-RADS score, missing histology results, and missing PSA values or prostate volume. Data from a total of 594 patients were analysed.

Data collection

For each patient, we recorded age, date of LATP and mpMRI scan, any previous prostate biopsies, family history of PC, smoking status, comorbidities, previous finasteride use, PSA volume (mL), PSA values (ng/mL), PI-RADS score on MRI report, and Gleason score in a Microsoft Excel sheet (Microsoft Corporation, Redmond, Washington, United States) [10,11]. mpMRI scans were interpreted by several consultant radiologists. Due to the retrospective nature of the study, documentation specifying the use of a consensus or standardised reporting approach was unavailable. Experienced urologists performed all procedures with a minimum of 24 systematic core biopsies obtained. PSAD was calculated using pre-MRI PSA values and prostate volume based on MRI scans. In cases where the prostate volume was reported as length, weight, and height, the ellipsoid formula was used to calculate the volume.

Data analysis

The highest PI-RADS score of each mpMRI scan and the highest Gleason score from the histology report were used for analysis. The PI-RADS scores were grouped as low risk (PI-RADS score 1-2), intermediate risk (PI-RADS score 3), and high risk (PI-RADS score 4-5). The histology results were grouped as benign, grade 1 (Gleason score 6), and grade ≥2 (Gleason scores 7, 8, 9, and 10). PSAD was categorised into low (<0.15 ng/mL^2^) and high (≥0.15 ng/mL^2^).

All data were analysed using IBM SPSS Statistics for Windows, Version 26.0 (Released 2019; IBM Corp., Armonk, New York, United States). Descriptive statistics were used to summarise the findings. Categorical data were displayed as frequency, and continuous data were described using mean and SD or median with range, where applicable. We performed two multinomial logistic regression models: (1) PSAD and PI-RADS main effects without covariates and (2) PSAD and PI-RADS main effects with covariates. A multinomial logistic regression model was used to assess the association of the PSAD category and PI-RADS group with histology outcome (1: benign; 2: Gleason grade 1; and 3: Gleason grade ≥2). Both main effects (high vs. low PSAD; PI-RADS 3 and PI-RADS 4-5 vs. PI-RADS 1-2), along with age as a confounding factor, were included. Benign histology, low PSAD, and PI-RADS 1-2 were used as the reference. Finasteride use (p=0.610) and previous biopsy status (p=0.052) were evaluated as potential confounders using chi-squared testing and were not found to be significantly associated with biopsy outcome; therefore, they were not included in the multinomial regression model. As PSAD is calculated using PSA and prostate volume, we did not include PSA and prostate volume in the regression model to avoid collinearity and overadjustment. To assess whether PI-RADS modified the association between PSAD and Gleason outcome, an interaction term (PSAD×PI-RADS) was initially introduced. However, the interaction model demonstrated instability with singular Hessian matrices and non-convergence due to sparse data within several predictor-outcome combinations. The interaction term was also not statistically significant (p=0.16). Hence, the interaction was removed, and the final model retained only main effects. Effect sizes were expressed as adjusted odds ratios (ORs) with 95% confidence intervals (CIs). Statistical significance was set as two-sided (p<0.05). Missing data was not imputed. Due to the audit nature of the study, sample size calculation was not required, and no formal hypothesis was made. The term clinically significant prostate cancer (csPCa) used in this study refers to patients with a Gleason grade ≥2.

Ethics statement

The study was approved by the Clinical Audit Team of Ysbyty Gwynedd (approval number: 2291). Given its retrospective design, informed consent was waived. All data were anonymised prior to analysis. As this was an audit of existing practice, no changes were made to clinical care pathways.

## Results

A total of 594 patient data were analysed. The mean age was 68.72, the median PSA value was 8.20 ng/mL, the median PSAD was 0.17 ng/mL^2^, and the median prostate volume was 48 mL. There were zero patients with PI-RADS 1, 10.61% had PI-RADS 2, 32.66% had PI-RADS 3, 30.98% had PI-RADS 4, and 25.76% had PI-RADS 5. The total histology outcomes were as follows: 30.13% benign and 69.87% malignant. Of the 415 malignant biopsy findings, 34.69%, 25.06%, 16.63%, 7.71%, and 15.9% had Gleason grades 1, 2, 3, 4, and 5, respectively. About 35.18% of patients had hypertension, 6.69% had diabetes, 0.57% had cerebrovascular accident (CVA), and 2.29% had ischemic heart disease (IHD). Around 4.2% of patients were on finasteride before biopsy, 9.93% of patients had a previous prostate biopsy, and 20.6% had a family history of PC. About 66.8% of patients presented with lower urinary tract symptoms before undergoing LATP. With regard to smoking status, 52.7% never smoked, 38.3% were ex-smokers, and 9.1% were current smokers (Table 1).

**Table 1:** Demographic variables This table summarises the baseline characteristics of 594 patients included in the study. Continuous variables are expressed as mean (SD) and categorical variables as numbers with percentages. Values are reported as the number of patients with percentages in parentheses. PSA: prostate-specific antigen; PSAD: prostate-specific antigen density; PI-RADS: Prostate Imaging Reporting and Data System; LUTS: lower urinary tract symptoms (frequency/urgency/dysuria/dribbling/haematuria)

Variables	Total (n=594)
Age, mean (SD)	68.72±7.73
PSA value (median)	8.20 ng/mL (range: 0.30-300.90)
PSAD, mean (SD)	0.17 ng/mL^2^ (range: 0.01-4.88)
Prostate volume (mL), median (range)	48.00 mL (range: 14.00-244.06)
PI-RADS score	
1	0 (0)
2	63 (10.61)
3	194 (32.66)
4	184 (30.98)
5	153 (25.76)
Prostate biopsy histology	
Benign	179 (30.13)
Malignant	415 (69.87)
Gleason grade	
1	144 (34.69)
2	104 (25.06)
3	69 (16.63)
4	32 (7.71)
5	66 (15.9)
Comorbidities	
Hypertension	184 (35.18)
Type 2 diabetes mellitus	35 (6.69)
Cardiovascular accidents	3 (0.57)
Ischemic heart disease	12 (2.29)
Clinical details	
Finasteride before biopsy	25 (4.2)
Previous prostate biopsy	59 (9.93)
LUTS symptoms	397 (66.8)
Family history of prostate cancer	121 (20.6)
Smoking history	
Never	306 (52.7)
Ex-smoker	223 (38.4)
Current smoker	52 (9)

Table 2 demonstrates the findings of PSAD with PI-RADS score in relation to the histology outcome. Of the total 179 patients with benign histology, 13.4% (24/179) had low PSAD (<0.15 ng/mL^2^) and low PI-RADS (score 1-2), 25.14% (45/179) had low PSAD and intermediate PI-RADS (score 3), and 20.11% (36/179) had low PSAD and high PI-RADS (score 4-5). Around 6.7% (12/179) had high PSAD (>=0.15ng/ml) and low PI-RADS, 21.78% (39/179) had high PSAD and intermediate PI-RADS, and 12.85% (23/179) had high PSAD and high PI-RADS.

**Table 2:** PSAD and PI-RADS distribution by histological outcome This table presents the distribution of patients according to PI-RADS score and PSAD (PSAD <0.15 ng/mL^2^ vs. ≥0.15 ng/mL^2^) stratified by histological outcome (benign histology, Gleason grade 1, and Gleason grade ≥2). PSAD: prostate-specific antigen density; PI-RADS: Prostate Imaging Reporting and Data System

Histological outcome	PI-RADS score	PSAD		Total
		<0.15 ng/mL^2^	≥0.15 ng/mL^2^	
Benign histology	Low risk (1-2)	24	12	36
	Intermediate risk (3)	45	39	84
	High risk (4-5)	36	23	59
	Total	105	74	179
Gleason grade 1	Low risk (1-2)	7	7	14
	Intermediate risk (3)	35	28	63
	High risk (4-5)	28	39	67
	Total	70	74	144
Gleason grade ≥2	Low risk (1-2)	0	13	13
	Intermediate risk (3)	14	33	47
	High risk (4-5)	34	177	211
	Total	48	223	271

Among the 144 patients with Gleason grade 1, 4.86% (7/144) had both low PSAD and low PI-RADS, 24.31% (35/144) had low PSAD and intermediate PI-RADS, and 19.44% (28/144) had low PSAD and high PI-RADS. Around 4.86% (7/144) had high PSAD and low PI-RADS, 19.44% (28/144) had high PSAD and intermediate PI-RADS, and 51.39% (74/144) had high PSAD and high PI-RADS.

In this group of 271 patients with Gleason grade ≥2, there were zero patients with low PSAD and low PI-RADS, 5.17% (14/271) with low PSAD and intermediate PI-RADS, and 12.55% (34/271) with low PSAD and high PI-RADS. About 4.8% (13/271) had high PSAD and low PI-RADS, 12.18% (33/271) had high PSAD and intermediate PI-RADS, and 65.07% (177/271) had high PSAD and high PI-RADS.

Table 3 demonstrates the multinomial regression model using the main effects of PSAD and PI-RADS with and without interaction on histology outcome. In patients with csPCa (Gleason ≥2 vs. benign), high PSAD (OR 5.93; 95% CI 3.74-9.40; p=0.00) and PI-RADS 4-5 were a strong predictor (OR 8.93; 95% CI 4.13-19.29; p=0.00). PI-RADS 3 was a predictor but did not reach significance (OR 1.59; 95% CI 0.74-3.43; p=0.23). For patients with Gleason grade 1 compared to the benign group, PSAD ≥0.15 showed a non‐significant trend toward increased odds (OR 1.46; 95% CI 0.93-2.28; p=0.09). PI-RADS 3 had a borderline association (OR 1.94; 95% CI 0.96-3.91; p=0.06). PI-RADS 4-5 was significantly associated with higher odds (OR 2.87; 95% CI 1.41-5.84; p=0.00). These findings indicate that both high PSAD and high PI-RADS independently are strongly associated with the presence of csPCa, with PI-RADS 4-5 also significantly predicting Gleason grade 1 disease.

**Table 3:** Multinomial logistic regression main-effects model of PSAD and PI-RADS for histological outcome This table presents the results of the multinomial logistic regression analysis evaluating the association between PSAD, PI-RADS score, and histological outcome (Gleason grade 1 and Gleason grade ≥2 vs. benign). In the model without interaction, high PSAD (≥0.15 ng/mL^2^) and higher PI-RADS scores (4-5) were significantly associated with malignant histology, particularly Gleason grade ≥2. *Statistically significant at α=0.05. PSAD: prostate-specific antigen density; PI-RADS: Prostate Imaging Reporting and Data System

Comparison	Predictor	OR	95% CI	P-value
Gleason 1 vs. benign (without covariates)	High PSAD (≥0.15)	1.46	0.93-2.28	0.09
	PI-RADS 3 vs. 1-2	1.94	0.96-3.91	0.06
	PI-RADS 4-5 vs. 1-2	2.87	1.41-5.84	0.004*
Gleason ≥2 vs. benign (without covariates)	High PSAD (≥0.15)	5.93	3.74-9.40	<0.001*
	PI-RADS 3 vs. 1-2	1.59	0.74-3.43	0.23
	PI-RADS 4-5 vs. 1-2	8.93	4.13-19.29	<0.001*
Gleason 1 vs. benign (with covariates)	High PSAD (≥0.15)	1.13	0.63-2.02	0.67
	PI-RADS 3 vs. 1-2	1.93	0.96-3.88	0.06
	PI-RADS 4-5 vs. 1-2	2.11	0.92-4.86	0.07
	PSAD×PI-RADS interaction	1.93	0.77-4.83	0.16
Gleason ≥2 vs. benign (with covariates)	High PSAD (≥0.15)	4.46	2.21-8.99	<0.001*
	PI-RADS 3 vs. 1-2	1.50	0.74-3.03	0.25
	PI-RADS 4-5 vs. 1-2	4.65	2.22-9.77	<0.001*
	PSAD×PI-RADS interaction	1.93	0.77-4.83	0.16

Applying age as the covariate in the regression model, high PSAD (OR 5.63; 95% CI 3.54-8.96; p=0.00) and high PI-RADS (OR 9.14; 95% CI 4.36-19.19; p=0.00) continue to show a high association in patients with csPCa (Gleason ≥2 vs. benign). In patients with Gleason grade 1, a high PI-RADS (OR 2.84; 95% CI 1.39-5.80; p=0.00) shows statistical significance; however, having a high PSAD (OR 1.42; 95% CI 0.91-2.23; p=0.13) does not. Age was shown to be statistically significant in patients with csPCa (OR 1.04; 95% CI 1.01-1.07; p=0.00) but not in patients with Gleason grade 1.

## Discussion

In our cohort of csPCa cases, PSAD ≥0.15 ng/mL^2^ was far more common than PSAD <0.15 (82% vs. 18%). All 13 patients with csPCa who had only low-suspicion PI-RADS 1-2 lesions on MRI still exhibited a PSAD ≥0.15. Sixty-five percent of all csPCa cases had the combination of high PSAD and high PI-RADS (4-5). This prevalence of elevated PSAD among csPCa patients across all PI-RADS categories mirrors findings from a literature review that noted higher rates of PSAD ≥0.15 in csPCa cases irrespective of PI-RADS group [12]. A study of biopsy-naive patients found that PSAD and PI-RADS were independent predictors of PC, including clinically significant disease [7]. Using a PSAD cutoff of ≥0.15 ng/mL^2^ yielded a specificity of 34%, sensitivity of 99%, positive predictive value of 59%, and negative predictive value of 96% for detecting csPCa [7]. The detection rate of csPCa ranged from 76% to 97% among patients who had either PI-RADS ≥4 with PSAD ≥0.15 or PI-RADS 3 with PSAD ≥0.30 ng/mL^2^. A further study reported that patients with both an elevated PSAD and a positive MRI have roughly double the risk of csPCa. At PSAD cutoffs of 0.10, 0.15, and 0.20 ng/mL^2^, the probability of csPCa approximately doubled with a positive MRI (rising from 26% to 57%, 30% to 65%, and 34% to 69%), respectively [9].

Our multinomial analysis reinforced the importance of both MRI findings and PSAD. Patients with PSAD >0.15 had approximately sixfold higher odds of csPCa, and those with a PI-RADS 4-5 lesion had nearly ninefold higher odds, with no significant interaction effect between these factors. This suggests that PSAD and PI-RADS provide largely additive (rather than synergistic) information in risk stratification. It is worth noting that we did not adjust for other potential confounders (such as age, PSA levels, and prostate volume); incorporating such variables in future studies could further refine these risk estimates. Our findings align with other reports in the literature [7,13]. One study found men with PI-RADS 4-5 lesions had significantly higher odds of csPCa, even when their PSAD was <0.10 ng/mL^2^ [13]. Conversely, in patients without a prior PC diagnosis and those on active surveillance with an equivocal or negative MRI, a higher incidence of csPCa was observed in those with PSAD ≥0.15 compared to those with lower PSAD levels. This trend was reflected in our cohort as well: of the 60 patients with PI-RADS ≤3 lesions who were diagnosed with Gleason ≥3+4 disease, approximately 77% had a PSAD ≥0.15.

Interestingly, among patients with benign biopsy results in our study, a small subset (12.8%, 23/179) presented with both a high PSAD and a high PI-RADS score. This finding underscores that even markedly elevated PSAD and suspicious MRI findings can sometimes occur in the absence of cancer. One study suggested that restricting biopsies to men with either a PI-RADS (biparametric MRI) score ≥4 or a PSAD ≥0.15 could avoid biopsies in 41% of men and reduce the overdiagnosis of insignificant cancers, at the cost of missing only about 5% of csPCa [14]. However, applying such criteria to our dataset would have resulted in a higher miss rate. In our cohort, 257 of 594 patients had PI-RADS 1-3 lesions, of whom 23.3% (60/257) were found to have csPCa. Similarly, 270 of 594 patients had PSAD <0.15, of whom 17.8% (48/270) were ultimately diagnosed with csPCa. These figures indicate that strictly omitting biopsy in patients with PI-RADS 1-3 or PSAD <0.15 would have missed a considerable fraction of significant cancers in our cohort.

PI-RADS 3 lesions have historically posed a management dilemma due to their intermediate risk profile. In our cohort, 24.2% (47/194) of PI-RADS 3 lesions were associated with csPCa on biopsy. The PSAD proved helpful in stratifying risk within this group: PI-RADS 3 lesions with PSAD <0.15 had a csPCa rate of about 15% (14/94), whereas those with PSAD ≥0.15 had a csPCa rate of 33% (33/100). These findings align with a meta-analysis that reported post-biopsy probabilities of approximately 12% and 33% for csPCa in patients with PI-RADS 3 lesions, depending on whether PSAD was <0.15 or >0.15, respectively [9]. In our regression model, PI-RADS 3 (compared to PI-RADS 1-2) was associated with only a borderline increase in odds of csPCa, but this did not reach statistical significance in either the low-grade (Gleason grade group 1) or high-grade (Gleason grade group ≥2) subsets. This underscores the uncertainty around PI-RADS 3 lesions and the need for additional risk stratifiers like PSAD or follow-up imaging in their management.

Although a PSAD cutoff of 0.15 ng/mL^2^ remains one of the most widely used thresholds for clinical decision-making, some authors have argued for adjusting this cutoff based on MRI quality and prostate volume. One study reported that a PSAD threshold of 0.15 ng/mL^2^ provides roughly 70% sensitivity and 70% specificity for detecting any PC, whereas raising the cutoff to 0.20 ng/mL^2^ improved specificity to 79% for detecting csPCa, with similar sensitivity [15]. A further study suggested that 0.15 ng/mL^2^ is an appropriate threshold only for lower-quality MRI settings, advocating that under routine high-quality MRI conditions, a higher cutoff (≥0.20 ng/mL^2^) may be more suitable [16].

In our cohort, 4.2% of patients were on finasteride at the time of biopsy. This is an important consideration because finasteride therapy lowers serum PSA levels and can thereby alter the calculated PSAD, potentially underestimating risk in those patients. Notably, one study reported that PSAD has limitations in very large prostates and found no direct correlation between PSA level and benign prostatic hyperplasia (BPH) tissue volume, implying that PSA (and thus PSAD) does not simply scale with prostate size [17]. Moreover, most men with PC have concomitant BPH, and when tumour volume is accounted for, an inverse correlation was observed between PSA levels and Gleason score. Corcoran et al. [18] further demonstrated that PSAD was the strongest predictor of tumour grade upgrading from initial biopsy to final prostatectomy pathology in men with Gleason 6 or Gleason 3+4 disease, although PSAD's predictive value diminished for cancers of higher Gleason scores.

Strengths and limitations

This study has several strengths. We analysed a relatively large cohort of patients with no history of PC, all of whom underwent mpMRI and PSAD assessment, strengthening the applicability of our findings. Additionally, multiple radiologists and urologists were involved in image interpretation and biopsy procedures for this cohort. The involvement of a broad range of practitioners, rather than a single expert, means our results are more likely to reflect those observed in routine clinical practice across different operators.

Several limitations of our study should be acknowledged. Firstly, the retrospective, single-centre study design may limit the generalisability of the results. All data were collected from one institution and retrospectively analysed, introducing potential selection bias and confounders. Secondly, our predictive modelling made certain assumptions that could impact its performance. For example, we narrowed our focus to PI-RADS and PSAD as key predictors; in doing so, we necessarily excluded some clinical variables (even those that were not statistically significant predictors of csPCa), which may affect the robustness of the model. This approach, while simplifying analysis, might overlook subtle interactions or contributions from omitted factors. Moreover, data on the time interval between PSA test, mpMRI, and biopsy were not collected, which is another potential source of bias.

Furthermore, we did not capture detailed data on the MRI acquisition protocols (such as sequence parameters) in our analysis. Variations in MRI technique or image quality and not accounting for them could affect PI-RADS scoring consistency and diagnostic performance. Moreover, we did not formally measure the experience level of the radiologists interpreting the MRIs. Differences in reader experience and interobserver variability can influence PI-RADS assessments and biopsy outcomes.

## Conclusions

This study demonstrates that both a high PSAD and a high PI-RADS score are strongly associated with csPCa. Notably, PSAD also adds discriminatory value in PI-RADS 3 lesions: even within this intermediate imaging category, a higher percentage of patients with csPCa had a higher PSAD. Prospective, multi-centre studies will be essential to validate these findings and address the noted limitations. Such future research should also use various cutoff points of PSAD to assess the implications of this on the association of PC.
